# Supplementary material for: Induced formation of primordial germ cells from zebrafish blastomeres by germplasm factors
Source: Nat Commun. 2023 Dec 14;14:7918. doi: 10.1038/s41467-023-43587-3 (PMC10721796; doi:10.1038/s41467-023-43587-3)
Supplement: Supplementary file 1 — Supplementary Information [file 41467_2023_43587_MOESM1_ESM.pdf]

Supplementary Information

Induced formation of primordial germ cells from zebrafish blastomeres by germplasm factors

Xiaosi Wang[1, 2, 3], Junwen Zhu[1, 2], Houpeng Wang[1, 3], Wenqi Deng[1, 2], Shengbo Jiao[1, 2], Yaqing Wang[1, 2, 3], Mudan He[1, 2, 3], Fenghua Zhang[1, 2], Tao Liu[1, 2], Yongkang Hao[1, 2], Ding Ye[1, 2, 3], Yonghua Sun[1, 2 ,3*]

[1]State Key Laboratory of Freshwater Ecology and Biotechnology, Key Laboratory of Breeding Biotechnology and Sustainable Aquaculture, Institute of Hydrobiology, Innovation Academy for Seed Design, Chinese Academy of Sciences, Wuhan 430072, China;
[2]College of Advanced Agricultural Sciences, University of Chinese Academy of Sciences, Beijing 100049, China
[3]Hubei Hongshan Laboratory, Wuhan 430070, China
*Correspondence to: Yonghua Sun, Email: yhsun@ihb.ac.cn

List of Supplementary Material:

Supplementary figures S1 to S5

Supplementary table S1 to S3

Supplementary Figures

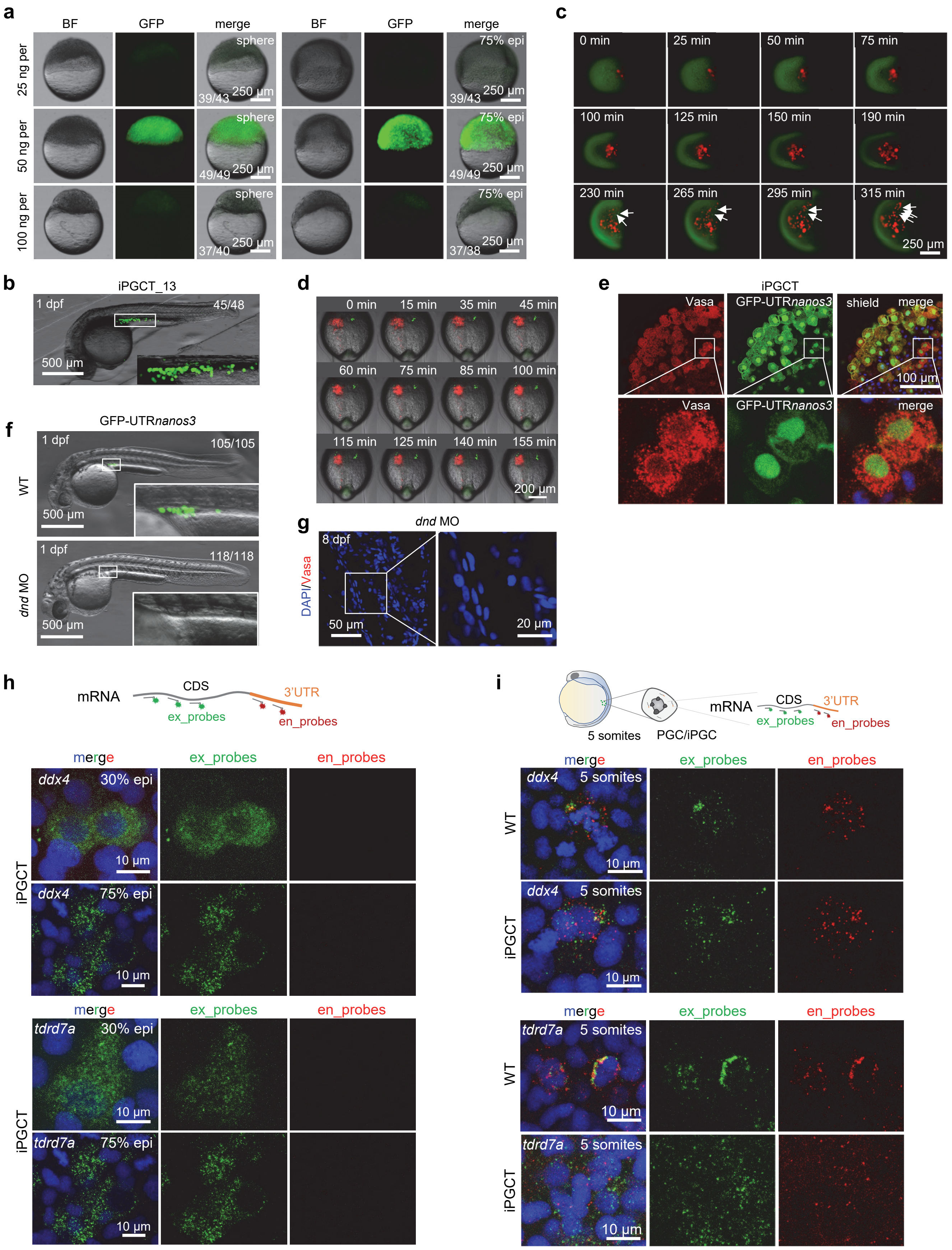

Supplementary Figure 1: iPGC has the same characteristics as PGC.

(a) Injection of high dose (100 pg for each factor) of 9GMs inhibited embryonic development, and Injection of low dose (25 pg for each factor) of 9GMs did not induce PGC well, while injection of moderate dose (50

pg for each factor) of 9GMs did not interfere with embryonic development and could effectively induce iPGCs. (b) iPGCs induced by 13 germplasm factors (iPGC13) could migrate to the genital ridges of the recipient embryo efficiently after cell transplantation. (c and d) About 10 iPGCs (To match the number of endogenous PGCs) were transplanted into *Tg(piwil1:egfp-UTRnanos3)* receptors, the endogenous PGCs (ePGC) were labeled green (White arrow marked), and the iPGCs were labeled by mCherry-UTR*nanos3*. iPGCs and ePGCs proliferation were tracked after transplantation. They all migrated to the genital ridge as the embryos development. (e) At shield stage, GFP-UTR*nanos3* positive cells showed Vasa antibody positive in embryos overexpressing germplasm factors. (f) Compared with the control, GFP-UTR*nanos3* was not detected after injection of *dnd* MO at 1 dpf. (g) Vasa antibody was not detected after injection of *dnd* MO at 8 dpf. (h and i) Probes were designed at different positions of mRNA, and single molecule in situ hybridization (smFISH) was used to detect neonatal mRNA. Probes for detecting overexpressed mRNA (ex_probes) and probes for neonatal mRNA (en_probes) were designed in the CDS and UTR regions of the mRNA, respectively. ex_probes signals of *tdrd7a* and *ddx4* were detected at 30% epiboly, 75% epiboly and 5-somite stages, while en_probes signals were detected only at 5-somite stage. A representative example of three replicate is shown.

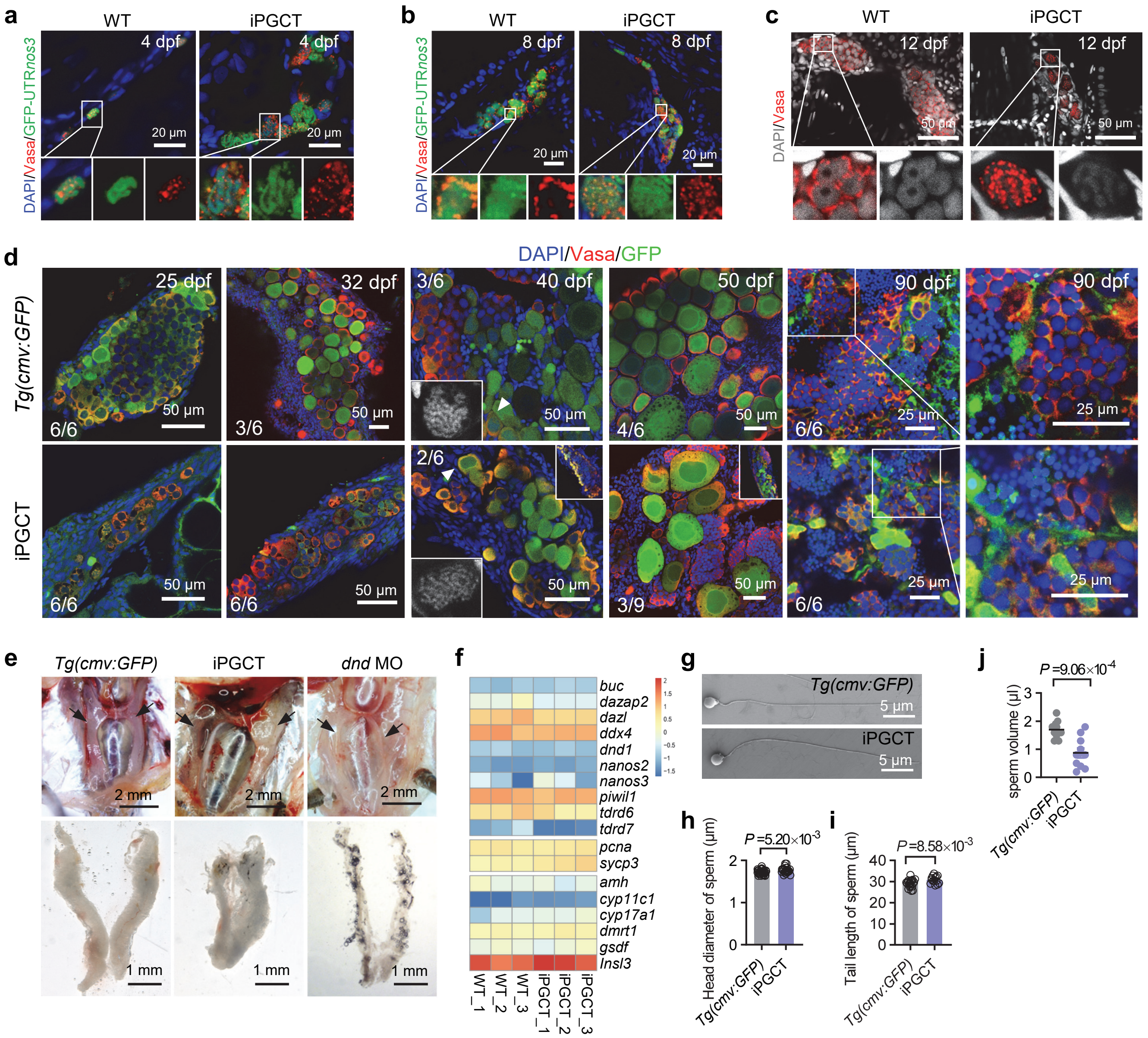

Supplementary Figure 2: The process of iPGCs proliferation and differentiation.

(a-d) Immunofluorescence imaging was used to track the process of iPGCs proliferation and differentiation. The enlarged image in Supplementary Figure 2d showed the chromatin status in the nucleus of the arrowheads. The iPGCT gonads of 40dpf and 50dpf showed the ovariform gonad, while the testicular form gonad was shown in the upper right corner of the image. (e) Morphology of the testes of *Tg(cmv:GFP)*, iPGC recombinant gonads and *dnd* MO. The figures below were the anatomical view of the gonads. The black arrow indicates the gonads. (f) Relative expression levels of germ cell-specific genes and gonadal somatic cell- specific genes in iPGCT testes. Relative expression levels (ΔCt) obtained by qRT-PCR. There were three technical replicates for each sample, and their average was used for calculation. In order to show the expression levels of multiple genes simultaneously, we used heat maps to show the relative expression levels of each gene. (g) Morphology of the sperm of *Tg(cmv:GFP)* and iPGCT. (h) Head diameter of sperm in *Tg(cmv:GFP)* and iPGCT. Each dot represents the head diameter of one sperm ($n \geq 22$). (i) Tail length of sperm in *Tg(cmv:GFP)* and iPGCT. Each dot represents the tail length of one sperm ($n \geq 14$). (j) Sperm volume of *Tg(cmv:GFP)* and iPGCT. Each dot represents the sperm volume of one fish

(n ≥ 10). A representative example of three replicate is shown. All data are presented as mean values ± SEM. Two-tailed Student's *t*-test was used to calculate the *P* values.

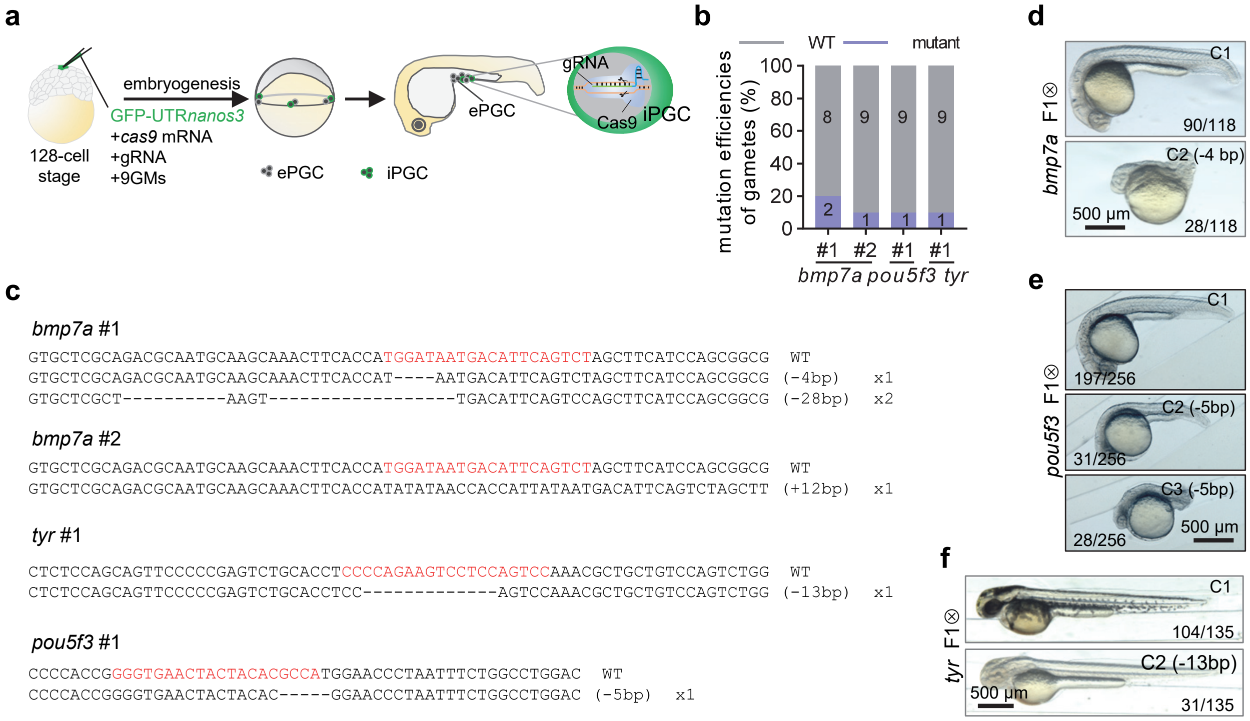

Supplementary Figure 3: The mutation types of gametes obtained by combining blastomere genome editing with iPGCs induction.

(a) Schematic representation of simultaneous genome editing and iPGC induction in any single blastomere at the 128-cell stage. These cells migrated to the genital ridge and eventually produced genome-edited gametes. (b) Mutation efficiencies of gametes. A total of 10 embryos obtained from hybridization of chimeras and wild-type were used to calculate gamete mutation efficiency. (c) 10 embryos obtained by mating chimeras with wild-type were sequenced. The type of mutation in each embryo reflects the type of mutation in the chimeric gametes. The red bases are the gRNA target sites. (d) The F1 *bmp7a*$^{-/+}$ adults derived from chimeras (*bmp7a* #1) produced F2 *bmp7a*$^{-/-}$ embryos showing severe dorsalization. (e) The F1 *pou5f3*$^{-/+}$ adults derived from chimeras (*pou5f3* #2) produced developmental defective F2 *pou5f3*$^{-/-}$ embryos (C2 and C3) with viable morphology. (f) The F1 *tyr*$^{-/+}$ adults derived from chimeras (*tyr* #2) produced F2 *tyr*$^{-/-}$ embryos without pigment. All F1 incross experiments have done for three replicates, and a representative example is shown.

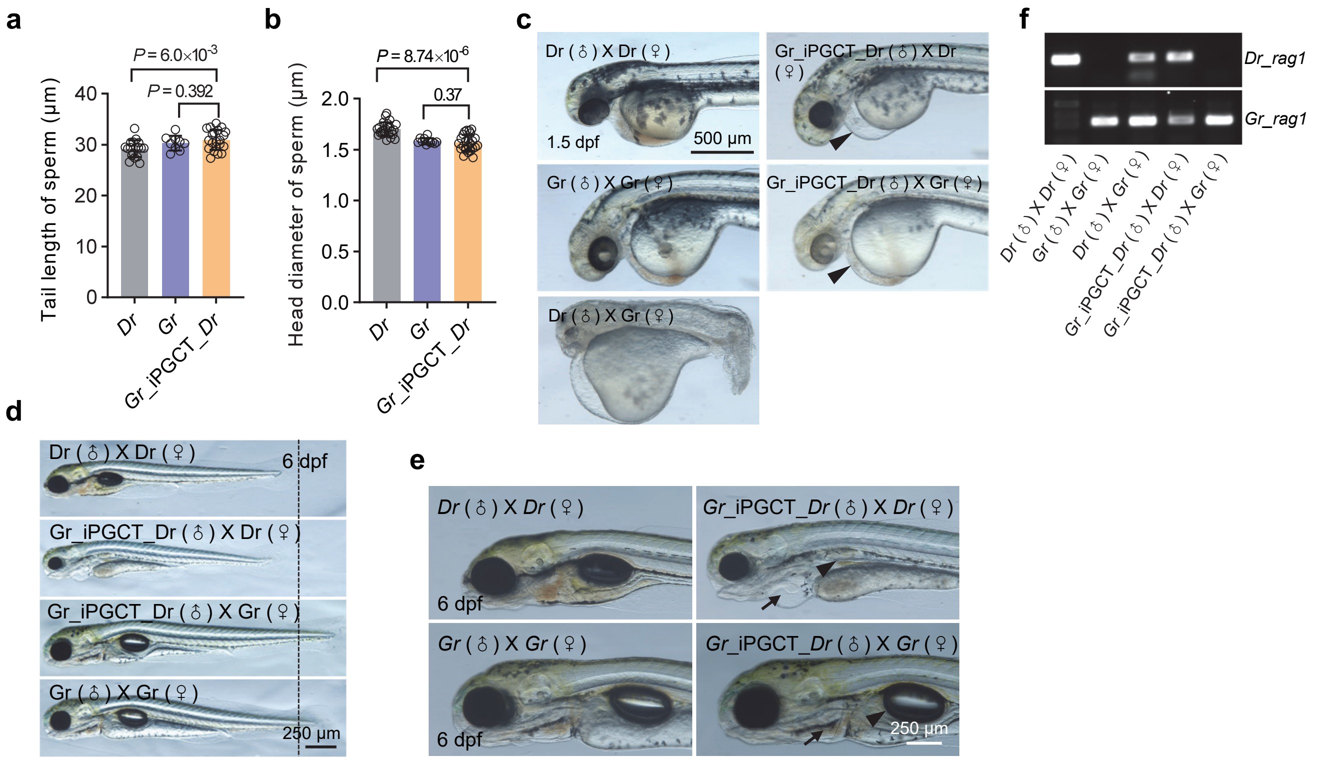

Supplementary Figure 4: *Gr*_iPGCT_*Dr* produces *Gr*-derived sperm.

(a) Tail length of sperm in *Gr*, *Dr* and *Gr*_iPGCT_*Dr*. Each dot represents the tail length of one sperm (n ≥ 9). (b) Head diameter of sperm in *Gr*, *Dr* and *Gr*_iPGCT_*Dr*. Each dot represents the head diameter of one sperm (n ≥ 11). (c) After mating between zebrafish (*Dr*) sperm and *Gobiocypris rarus* (*Gr*) eggs, the embryos show severe developmental defects at 1.5 dpf. After mating between *Gr*_iPGCT_*Dr* sperm and *Gr* egg, the embryo development was similar to that of *Gr*. However, when *Gr*_iPGCT_*Dr* sperm mated with *Dr* eggs, the embryos were similar to zebrafish and showed slight heart development defects. The arrowhead indicates the position of the heart. (d-e) After fertilization between *Gr*_iPGCT_*Dr* sperm and zebrafish egg, the body length of the embryo was similar to that of *Dr* at 6 dpf, and it showed small eyes, uninflated swim bladder, and heart development defects. However, after fertilization of *Gr*_iPGCT_*Dr* sperm and *Gr* egg, the embryo development was similar to that of *Gr*. (f) Molecular examination showed that sperm produced by *Gr*_iPGCT_*Dr* was derived from donor *Gr.* All data are presented as mean values ± SEM. Two-tailed Student's *t*-test was used to calculate the *P* values. A representative example of three replicate is shown.

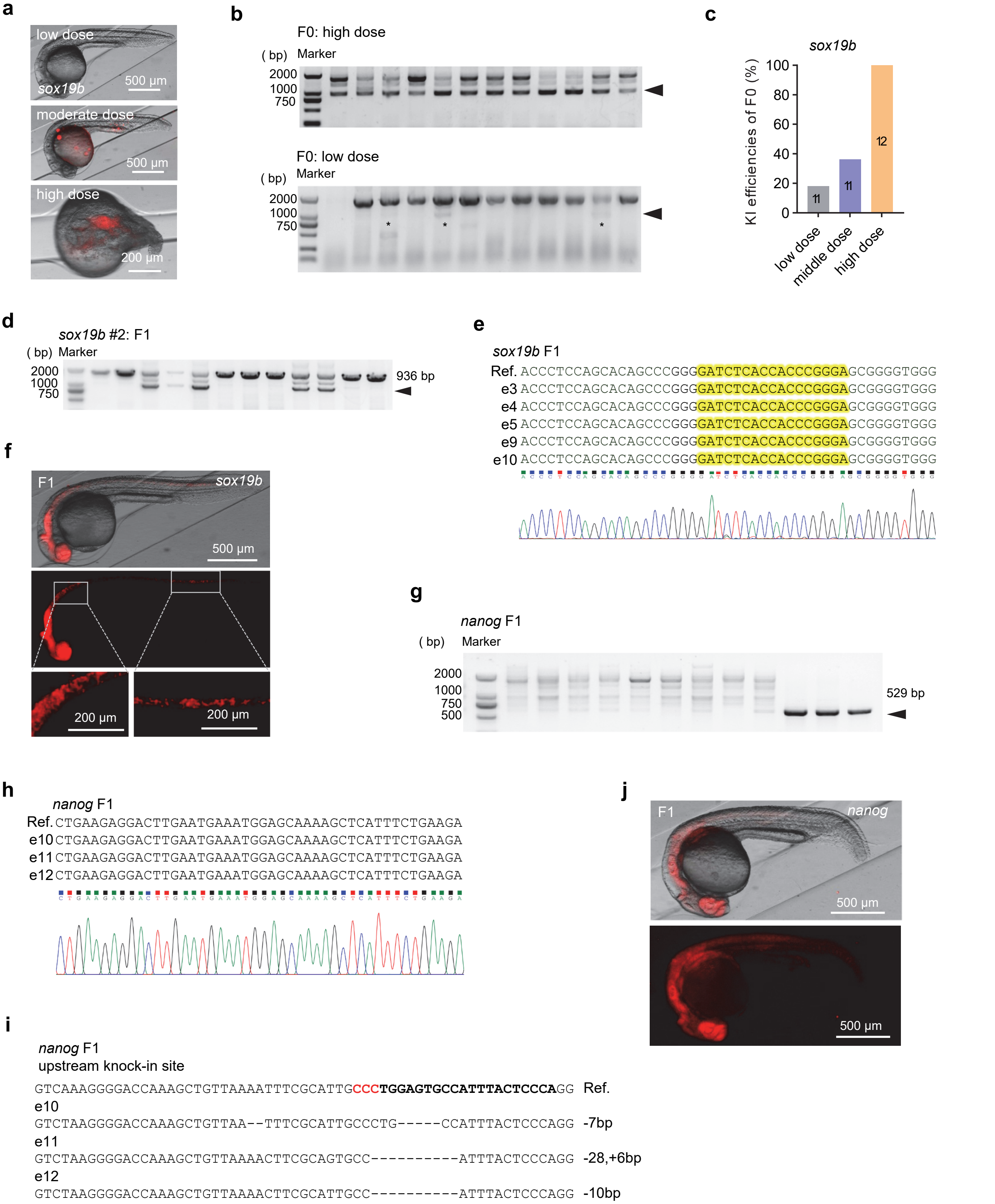

Supplementary Figure 5: Genome editing combined with iPGCT greatly improved the KI efficiency of gametes.

(a) With the increase of KI element concentration, the embryos appeared more and more serious abnormality rate. Low dose (50 pg gRNA, 50 pg plasmid, 500 pg *cas9* mRNA); moderate dose (100 pg gRNA, 100 pg plasmid, 1000 pg *cas9* mRNA); high dose (200 pg gRNA, 200 pg plasmid, 1000 pg *cas9*

mRNA). (b) Single embryos were used to evaluate the efficiency of knock-in events. The arrowhead indicated the positive bands (936 bp), and the asterisk represented the positive embryos. (c) Knock-in efficiency of F0 embryos at different doses. The number on the column represented the total number of embryos tested. (d) F0 was mated with the wild type, and 12 embryos were selected for identification one by one to evaluate the KI efficiency of F0 gamete. The arrowhead indicated the positive bands (936 bp). (e) Sequencing results of positive bands. gRNA target locations are marked in yellow. (f) Image of a positive progeny from a wild fish mated with F0 (#2). (g and h) 12 embryos were selected for identification to evaluate the KI efficiency of F0 gamete. Primers bind to the inserted myc and mCherry fragments. The arrowhead indicated the positive bands (529 bp). (h) Partial sequence of *nanog* positive band detection. (i) Sequence of F1 positive embryo knock-in site. The bolded base is the location of the gRNA target sequence, and the red base is the location of PAM. (j) Image of a positive progeny from a wildtype fish mated with F0. A representative example of three replicate is shown.

Supplementary Tables

Supplementary Table 1: qPCR primer sequence

primer name	sequence
ddx4-qPCR-F	TGCAGGACCCAAGGTTGTTT
ddx4-qPCR-R	GCTTTTGGAGGATTGCTGCC
piwil1-qPCR-F	TGACATAACAGATGGCAACCA
piwil1-qPCR-R	GCCCTCTCTCTGTTCAGGACT
nanos3-qPCR-F	ATGGCTTTTTCTCTTCTCCAAT
nanos3-qPCR-R	GTGTTCTGCTCCGGTGAGTC
dnd1-qPCR-F	TGATTCCTCAACCCACCATAA
dnd1-qPCR-R	TGGACTTCATATTGCGGAGA
buc-qPCR-F	CAAGTTACTGGACCTCAGGATC
buc-qPCR-R	GGCAGTAGGTAAATTCGGTCTC
dazap2-qPCR-F	GTCTCAGTATCCAGACGCCC
dazap2-qPCR-R	CGGATAATACGCCATCGGGA
tdrd6-qPCR-F	GACACAGCTCCCCGTGATAAG
tdrd6-qPCR-R	CATCTCCATGAAGCGTGCC
tdrd7a-qPCR-F	GATGTGTTTTGCTGGCGTGT
tdrd7a-qPCR-R	GACAAGCGAGGTTTGACGTT
dazl-qPCR-F	ATCGTCAGGGTTTTCCGTCC
dazl-qPCR-R	ATCAATACCGCCGACGAACA
nanos2-qPCR-F	GTTTCCTGATGTGGCGGGAT
nanos2-qPCR-R	GGTCCTGATGAAACCCTCCG
gsdf-qPCR-F	AGAGCCACAGCAGAGAGCAG
gsdf-qPCR-R	ACCTGAGAGGAGCGTCTGCA
amh-qPCR-F	TCGATGGATGATAACAGGCGAA
amh-qPCR-R	GGCTTGATCGTCGTACTGCT
cyp11c1-qPCR-F	CCATATACAGAGAGCACCTGG
cyp11c1-qPCR-R	AGACGGTCAGCACGCCACT
cyp17a1-qPCR-F	GGCCACGGACTGTTACAACAG
cyp17a1-qPCR-R	GGCTTTCAGTCAACACTTCACAC
dmrt1-qPCR-F	CTCCAACCAACCTAGGCAGTC
dmrt1-qPCR-R	ATGGAGTGGGCTGGTAAAGG
insl3-qPCR-F	TCGCATCGTGTGGGAGTTT
insl3-qPCR-R	GCACAACGAGGTCTCTATCCA
sycp3-qPCR-F	CGGATCTGACGAAGACACGA
sycp3-qPCR-R	TGCTGATGTCCGCACCAAA
pcna-qPCR-F	CTGGTCTTTGAAACGCTCAATCA
pcna-qPCR-R	CGGCATCTTCACCACACAAC

Supplementary Table 2: smFISH primer sequence

primer name	sequence
tdrd7a-P1-F-594 UTR	CCTCgTAAATCCTCATCAAAacttgttttttcgccgttaatgcg
tdrd7a-P1-R-594 UTR	tcactcatcctgccgatttctctttAAATCATCCAgTAAACCgCC
tdrd7a-P2-F-594 UTR	CCTCgTAAATCCTCATCAAActgagagtatcaagcatctctgctg
tdrd7a-P2-R-594 UTR	cagcaaatgttctgggaaagtgcagAAATCATCCAgTAAACCgCC
tdrd7a-P3-F-594 UTR	CCTCgTAAATCCTCATCAAAacatgacatgcgtccaccaaacacg
tdrd7a-P3-R-594 UTR	aggaacgtacaaaaataaattgagcAAATCATCCAgTAAACCgCC
tdrd7a-P4-F-594 UTR	CCTCgTAAATCCTCATCAAAgcactgacatattattattaacggc
tdrd7a-P4-R-594 UTR	tagatgttcaggtaacaatttattaAAATCATCCAgTAAACCgCC
ddx4-P1-F-594 UTR	CCTCgTAAATCCTCATCAAAaggtggacacgcgtgaagagcctga
ddx4-P1-R-594 UTR	agactttttttcagaagagccggtaAAATCATCCAgTAAACCgCC
ddx4-P2-F-594 UTR	CCTCgTAAATCCTCATCAAActgaaatggtattgaagaagctcgc
ddx4-P2-R-594 UTR	tttagtcattcaacattaacaaataAAATCATCCAgTAAACCgCC
ddx4-P3-F-594 UTR	CCTCgTAAATCCTCATCAAAgaggtcaaacctacagcattcaatg
ddx4-P3-R-594 UTR	aaggtattagtcttgattatcgtccAAATCATCCAgTAAACCgCC
ddx4-P4-F-594 UTR	CCTCgTAAATCCTCATCAAAgttctatactctggcttcaagacgg
ddx4-P4-R-594 UTR	aaggtacatctgcaaaacaagtttcAAATCATCCAgTAAACCgCC
tdrd7a-P1-F-cy5 CDS	CCTCAACCTACCTCCAACAAtgtgtgctgtctcctcacacacgcc
tdrd7a-P1-R-cy5 CDS	tcttctgccgggcgaccagctgtgcATTCTCACCATATTCgCTTC
tdrd7a-P2-F-cy5 CDS	CCTCAACCTACCTCCAACAAcctgaaccaactcaacatctgctcg
tdrd7a-P2-R-cy5 CDS	acttctgcaaaagctgtttgattcgATTCTCACCATATTCgCTTC
tdrd7a-P3-F-cy5 CDS	CCTCAACCTACCTCCAACAActatattgttgctgcctggcttctc
tdrd7a-P3-R-cy5 CDS	ccaagacaggatacaccagacggtcATTCTCACCATATTCgCTTC
tdrd7a-P4-F-cy5 CDS	CCTCAACCTACCTCCAACAAactgcagagtcttcagaacaactgg
tdrd7a-P4-R-cy5 CDS	ccagcagcgatcgaccgacagctaaATTCTCACCATATTCgCTTC
tdrd7a-P5-F-cy5 CDS	CCTCAACCTACCTCCAACAAacaagatgtctaacacgtgactgcc
tdrd7a-P5-R-cy5 CDS	gagaagcaggaagccccagatccaaATTCTCACCATATTCgCTTC
ddx4-P1-F-cy5 CDS	CCTCAACCTACCTCCAACAAacttcctccagccattttggaacta
ddx4-P1-R-cy5 CDS	cctgttgtgccatgagcactgaaggATTCTCACCATATTCgCTTC
ddx4-P2-F-cy5 CDS	CCTCAACCTACCTCCAACAAgagcaattcaagcagctggtccctc
ddx4-P2-R-cy5 CDS	cattgtgcgctcattacctgttgctATTCTCACCATATTCgCTTC
ddx4-P3-F-cy5 CDS	CCTCAACCTACCTCCAACAAtcactgaacttgctggctgccacac
ddx4-P3-R-cy5 CDS	acgattatggcctcaggctcctgtaATTCTCACCATATTCgCTTC
ddx4-P4-F-cy5 CDS	CCTCAACCTACCTCCAACAAcgaaaactacccccgaaaccacctc
ddx4-P4-R-cy5 CDS	tcattaccaccatcacggaaacctcATTCTCACCATATTCgCTTC

Supplementary Table 3: gRNA sequence

gRNA name	Sequence (5’-3’)
bmp7a-gRNA	AGACTGAATGTCATTATCCA
tyr-gRNA	GGACTGGAGGACTTCTGGGG
pou5f3-gRNA	GGGTGAACTACTACACGCCA
mpx-gRNA	CCTTCAAAGATCACCCTCCA
sox19b-gRNA	TGCCCGGAGGAGACATGCCC
nanog-gRNA	TGGGAGTAAATGGCACTCCA
