# Supplementary material for: Induced formation of primordial germ cells from zebrafish blastomeres by germplasm factors
Source: Nat Commun. 2023 Dec 14;14:7918. doi: 10.1038/s41467-023-43587-3 (PMC10721796; doi:10.1038/s41467-023-43587-3)
Supplement: Supplementary file 3 — Description of Additional Supplementary Information [file 41467_2023_43587_MOESM3_ESM.pdf]

Supplementary Movie 1: iPGCs migrated to the genital ridge after iPGC transplantation.
GFP-UTR*nanos3* was used to label iPGCs. 9GMs were injected at 1-cell stage, and at 30% epiboly, 10-20 cells were transplanted into the recipient embryos whose endogenous germ cells were removed.

Supplementary Movie 2: iPGCs underwent rapid division during their migration to the genital ridge.
At 30% epiboly stage, about 10 iPGCs labled with mCherry-UTR*nanos3* were transplanted into *Tg(piwil1:egfp-UTRnanos3)* embryos. The white arrows indicate the endogenous PGCs labeled with *Tg(piwil1:egfp-UTRnanos3)*.

Supplementary Movie 3: The video of iPGC transplantation.
This video shows the operation of the iPGC transplantation. In iPGC transplantation, donor cells can be derived from cells anywhere in the embryo, not only restricted to cells at the margin of the embryo. 10-20 cells were transplanted into the recipient embryo whose endogenous germ cells were removed. Therefore, a large number of iPGCs can be provided as transplantation donors, greatly improving the efficiency and success rate of iPGC transplantation.

Supplementary Movie 4: iPGC and ePGC migrated to the genital ridge simultaneously.
The iPGCs were labeled with the mCherry-UTR*nanos3*. Endogenous PGCs were labeled with *Tg(piwil1:egfp-UTRnanos3)*. iPGCs and ePGCs migrated to the genital ridge simultaneously.

Supplementary Movie 5: iPGCs migrated to the genital ridge after microinjection in one blastula at 128-cell stage.
iPGCs were induced by injecting 9GMs into a single blastomere at the animal pole of the 128-cell stage embryos. GFP-UTR*nanos3* was used to label iPGCs.

Supplementary Movie 6: *Gr*_iPGC migrated to the genital ridge of *Dr* after transplantation.
iPGCs of *Gobiocypris rarus* (*Gr*) were induced by injection of zebrafish-derived 9GMs at 1-cell stage. GFP-UTR*nanos3* was used to label iPGCs. 10-20 iPGCs of *Gr* were transplanted into zebrafish embryos whose ePGC had been removed.
