# Supplementary material for: Induced formation of primordial germ cells from zebrafish blastomeres by germplasm factors
Source: Nat Commun. 2023 Dec 14;14:7918. doi: 10.1038/s41467-023-43587-3 (PMC10721796; doi:10.1038/s41467-023-43587-3)
Supplement: Supplementary file 10 — Reporting Summary [file 41467_2023_43587_MOESM10_ESM.pdf]

nature portfolio

Corresponding author(s): Yonghua Sun

Last updated by author(s): Oct 8, 2023

Reporting Summary

Nature Portfolio wishes to improve the reproducibility of the work that we publish. This form provides structure for consistency and transparency in reporting. For further information on Nature Portfolio policies, see our Editorial Policies and the Editorial Policy Checklist.

Please do not complete any field with "not applicable" or n/a. Refer to the help text for what text to use if an item is not relevant to your study. For final submission: please carefully check your responses for accuracy; you will not be able to make changes later.

Statistics

For all statistical analyses, confirm that the following items are present in the figure legend, table legend, main text, or Methods section.

n/a	Confirmed	
☐	☒	The exact sample size (*n*) for each experimental group/condition, given as a discrete number and unit of measurement
☐	☒	A statement on whether measurements were taken from distinct samples or whether the same sample was measured repeatedly
☐	☒	The statistical test(s) used AND whether they are one- or two-sided<br>*Only common tests should be described solely by name; describe more complex techniques in the Methods section.*
☒	☐	A description of all covariates tested
☒	☐	A description of any assumptions or corrections, such as tests of normality and adjustment for multiple comparisons
☐	☒	A full description of the statistical parameters including central tendency (e.g. means) or other basic estimates (e.g. regression coefficient) AND variation (e.g. standard deviation) or associated estimates of uncertainty (e.g. confidence intervals)
☐	☒	For null hypothesis testing, the test statistic (e.g. *F*, *t*, *r*) with confidence intervals, effect sizes, degrees of freedom and *P* value noted<br>*Give P values as exact values whenever suitable.*
☒	☐	For Bayesian analysis, information on the choice of priors and Markov chain Monte Carlo settings
☒	☐	For hierarchical and complex designs, identification of the appropriate level for tests and full reporting of outcomes
☒	☐	Estimates of effect sizes (e.g. Cohen's *d*, Pearson's *r*), indicating how they were calculated

Our web collection on statistics for biologists contains articles on many of the points above.

Software and code

Policy information about availability of computer code

Data collection	iPGCT transplantation was performed using CellTram 4r Oil (Eppendorf) instruments, the transplanting needle was made using Flaming/ Brown type micropipette puller (P-100, Sutter) and the needle was polished using Micro Grinder (EG-400, NARISHIGE). Testis and early embryos were imaged using a fluorescence stereomicroscope (Axio Zoom.V16, Zeiss), while sections and primitive gonads were imaged using Confocal microscope (SP8, Leica). The migration of iPGCs was tracked using a fluorescence microscope (CTR6500, Leica). The schematic drawing were drawn using Microsoft Office PowerPoint.
Data analysis	Fiji was used for image analysis. Graphs were made via GraphPad v8.

For manuscripts utilizing custom algorithms or software that are central to the research but not yet described in published literature, software must be made available to editors and reviewers. We strongly encourage code deposition in a community repository (e.g. GitHub). See the Nature Portfolio guidelines for submitting code & software for further information.

Data

Policy information about availability of data

All manuscripts must include a data availability statement. This statement should provide the following information, where applicable:

- Accession codes, unique identifiers, or web links for publicly available datasets
- A description of any restrictions on data availability
- For clinical datasets or third party data, please ensure that the statement adheres to our policy

The authors declare that the data supporting the findings of this study can be found in the article and Supplementary Information files. All other relevant data supporting the findings of this study are available upon request from the corresponding author. This article provides the source data.

Field-specific reporting

Please select the one below that is the best fit for your research. If you are not sure, read the appropriate sections before making your selection.

☒ Life sciences ☐ Behavioural & social sciences ☐ Ecological, evolutionary & environmental sciences

For a reference copy of the document with all sections, see nature.com/documents/nr-reporting-summary-flat.pdf

Life sciences study design

All studies must disclose on these points even when the disclosure is negative.

Sample size	All samples that met the requirements were counted and presented in the manuscript.
Data exclusions	No data were excluded for analysis.
Replication	At least three independent repeated experiments were carried out and a representative example of three replicate is shown.
Randomization	No specific randomization methods was used, the samples were carried out according to the specific experimental methods.
Blinding	No blinding were used.

Reporting for specific materials, systems and methods

We require information from authors about some types of materials, experimental systems and methods used in many studies. Here, indicate whether each material, system or method listed is relevant to your study. If you are not sure if a list item applies to your research, read the appropriate section before selecting a response.

Materials & experimental systems

n/a	Involved in the study	
☐	☒	Antibodies
☒	☐	Eukaryotic cell lines
☒	☐	Palaeontology and archaeology
☐	☒	Animals and other organisms
☒	☐	Clinical data
☒	☐	Dual use research of concern
☒	☐	Plants

Methods

n/a	Involved in the study	
☒	☐	ChIP-seq
☒	☐	Flow cytometry
☒	☐	MRI-based neuroimaging

Antibodies

Antibodies used	Anti-Vasa, anti-Sycp3 and anti-PCNA were against the antigens, and purified using antigen-affinity chromatography by our lab. These antibodies were conjugated with Alexa Fluor 488, 568 and 680 on demand using Alexa Fluor® Antibody Labeling Kits according to the manufacturer's manual (Thermo Fisher).
Validation	The antibodies used are all proven by a recent study (Ye et al., 2023).

Animals and other research organisms

Policy information about studies involving animals; ARRIVE guidelines recommended for reporting animal research, and Sex and Gender in Research

Laboratory animals	Both zebrafish and ***Gobiocypris rarus*** used in this study were raised in the China zebrafish Resource Center (CZRC). Our experiments were carried out using 3-month-old zebrafish from wild-type stocks with AB genetic background and transgenic line Tg(cmv:GFP) and Tg(cmv:mCherry). iPGC induction was performed in zebrafish embryos at 1-cell stage, and iPGC transplantation was performed when the embryos developed to sphere stage.
Wild animals Reporting	This study did not involve wild animals.
on sex Field-collected	No sex-specific fish were selected for the experiment. The resulting offspring are all male.
samples Ethics	All animal experiments were conducted according to the standard animal guidelines approved by the Animal Care Committee of the University of Chinese Academy of Sciences and the Institute of Hydrobiology, Chinese Academy of Sciences.
oversight	All animal experiments are supervised by the Animal Care Committee of the University of Chinese Academy of Sciences.

Note that full information on the approval of the study protocol must also be provided in the manuscript.

This checklist template is licensed under a Creative Commons Attribution 4.0 International License, which permits use, sharing, adaptation, distribution and reproduction in any medium or format, as long as you give appropriate credit to the original author(s) and the source, provide a link to the Creative Commons license, and indicate if changes were made. The images or other third party material in this article are included in the article's Creative Commons license, unless indicated otherwise in a credit line to the material. If material is not included in the article's Creative Commons license and your intended use is not permitted by statutory regulation or exceeds the permitted use, you will need to obtain permission directly from the copyright holder. To view a copy of this license, visit http://creativecommons.org/licenses/by/4.0/
